# Supplementary material for: Intimate partner violence (IPV) with miscarriages, stillbirths and abortions: Identifying vulnerable households for women in Bangladesh
Source: PLoS One. 2020 Jul 28;15(7):e0236670. doi: 10.1371/journal.pone.0236670 (PMC7386588; doi:10.1371/journal.pone.0236670)
Supplement: Attachment — Submitted filename: Comments.docx [file pone.0236670.s001.docx]

Comments

This topic is important in terms of developing regions as IPV is prevalent in these regions. After going through the article, I am wondering at some points.

Comments:

1. The introduction section is redundant in some aspect. However, the study did not add previous studies regarding the subject matter. What is the actual gap of the study is missing?

2. The theoretical framework is resourceful in terms of theories. How it linked to the present studies, the figure 1 does not provide enough information.

3. In methodology section, how outcome variables were calculated and measure need to explain. In statistical analysis section, model fit information need to add.

4. In Table 1, better to report the Phi coefficient and Cramer's V (Strength of association) along with the p-value obtained from the Chi-square test.

5. Repetition of some information in introduction, discussion and conclusion section might degrade the quality.

6. This study is based on the data of BDHS 2007. What this study adds in terms of current policy implication while Bangladesh is doing better over most of the selected indicators.
